# Safety Evaluation of Yeasts With Probiotic Potential

**DOI:** 10.3389/fnut.2021.659328

**Published:** 2021-05-21

**Authors:** Pilar Fernández-Pacheco, Inés María Ramos Monge, Mónica Fernández-González, Justa María Poveda Colado, María Arévalo-Villena

**Affiliations:** ^1^Analytical Chemistry and Food Technology Department, Faculty of Environmental Science and Biochemistry, Castilla-La Mancha University, Toledo, Spain; ^2^Analytical Chemistry and Food Technology Department and Instituto Regional de Investigación Científica Aplicada (IRICA), Faculty of Chemical Sciences and Technologies, Castilla-La Mancha University, Ciudad Real, Spain

**Keywords:** probiotic yeasts, antimicrobial resistance, enzymatic activities, safety, biogenic amines

## Abstract

This work has evaluated the safety aspects of 20 yeast strains, isolated from food environments, selected in previous works due to their probiotic potential. Among the different strains, there are *Saccharomyces* and non-*Saccharomyces* yeasts. Before safety evaluation, differentiation of *Saccharomyces cerevisiae* strains was done by PCR amplification of inter-δ region with pairs of primers δ2-12 and δ12-21, which showed that they were all different from each other and also had different profiles to *Saccharomyces boulardii* (the only commercial probiotic yeast). The non-*Saccharomyces* ones were already known. The evaluation tests carried out were antibiotic and antifungal resistance, production of biogenic amines, deconjugation activity of bile salts, and different enzymatic activities: coagulase, deoxyribonuclease, hemolysin, proteolytic, and phospholipase. None of the studied strains demonstrated coagulase, hemolytic or DNase capacity (clear virulence factors), although all of them showed protease activity, some showed phospholipase activity, and half of the yeasts were capable of conjugating bile salts. Regarding antimicrobial compounds, all were resistant to antibiotics but showed sensitivity to the antimycotics used. Nevertheless, only one strain of *Hanseniaspora osmophila* was excluded for use in the food industry, due to its high production of tyramine.

## Introduction

In Spain there is currently only one yeast strain marketed as a probiotic (CNCM I-745, Ultra-Levura), belonging to *Saccharomyces boulardii* species, although in recent years there have been numerous research studies about probiotic capability in yeasts (1–6). These studies have shown the good probiotic potential of strains of *Saccharomyces cerevisiae, Hanseniaspora osmophila, Lachancea thermotolerans, Metschnikowia ziziphicola*, and *Pichia kudriavzevii*, among others.

Researchers focus their attention on yeasts as they could offer a good alternative to probiotic bacteria for diversification of products. Besides, they have some advantages, for example, their translocation has never been reported and also, they limit the development of antibiotic resistance, since they cannot spread the genes (7).

A search in PubMed or WOS (September, 2020) with the keywords “probiotic yeasts” returns 1,078 and 1,370 articles from the last 20 years, which is still very few if comparing with “probiotic bacteria” (19,050 and 14,480 articles found). Most of the published works focus on the evaluation of adapting and colonizing the gastrointestinal tract, as well as its possible mechanisms of action which exert health-promoting effects (8, 9). Nevertheless, there is very little information about their safety.

Yeasts are widely distributed in food, especially *S. cerevisiae*, which is responsible for the fermentation processes in beer, cider, sake, wine and bread making, among others. Until few years ago, it was considered as a safe microorganism (10). But due to an increasing number of reports about yeasts in superficial and life-threatening systemic diseases, the status of *Saccharomyces* sp. has changed from a group which is generally recognized as safe (GRAS) to a group of opportunistic pathogens of low virulence (11), although some authors attribute it to their wide distribution and use (10). The main cases correspond to fungemias, although it is also important to note that the number of cases is very low and that most of them are associated with immunosuppressed people. However, it is still unclear whether some *S. cerevisiae* strains are more likely to cause infections than others (11).

Regarding non-*Saccharomyces* species (except *Candida albicans* which is classified as a widely studied opportunistic pathogen), some of them, such as *Debaryomyces hansenii, Kluyveromyces lactis*, and *Kluyveromyces fragilis* among others, were approved by the EFSA (European Food Safety Authority) and included on the list of “Qualified Presumption of Safety” (QPS) i.e., microorganisms assumed safe (12, 13). However, there is still more uncertainty and confusion than with *Saccharomyces*. In any case, when infectious problems have been attributed to no pathogenic yeasts, the cases have been considered as biosecurity level I, with level IV being the maximum (10).

Therefore, in the search for potential probiotic yeasts, safety aspects should be considered including specifications such as origin, identity, and lack of harmful activities (14). Virulence is probably the most relevant factor, and it is the most repeated criterion in the bibliography. The DNase activity could be important for promoting DNA degradation of other microorganisms present in microenvironments (15, 16), while the hemolytic activity could be responsible for the occurrence of anemia and/or edema in the host. Another putative virulence factor is coagulase activity, which could cause blood clotting due to the coagulase enzyme attaching to plasma fibrinogen and causing the conversion of fibrinogen to fibrin (17). On the other hand, it is also necessary the evaluation of yeast growth in pseudohyphal form, because it is associated to some pathogens with the ability to invade epithelial and endothelial cells and cause damage to the tissues allowing access to the bloodstream (18). An assessment of antimicrobial resistance was also carried out, since this characteristic jeopardizes the efficacy of prevention and treatment against illnesses caused by mycotic infections (19). In addition to these virulence factors, other attributes that may contribute to pathogenicity are the production of extracellular phospholipase and protease (20, 21). When these are in high concentrations, they can increase the ability of certain microorganisms to colonize and penetrate the host tissue, causing tissue damage in the host cells by breaking the membranes of the epithelial cells (22). Nevertheless, there are also some other important aspects that have to be considered. For example, the ability to detoxify bile salt by producing bile salt hydrolase (BSH) enzyme activity (23) or the decarboxylation of certain amino acids and production of biogenic amines (BAs), which are responsible for adverse effects and are involved in several pathogenic syndromes. Histamine and tyramine the most dangerous BAs, causing the alterations known as “scombroid fish poisoning” and “cheese reaction,” respectively (24, 25). Histamine levels in fish have been considered as indicators of fast spoilage of fish, giving information on early microbial spoilage of seafood. Fifty ppm of histamine is considered as the chemical index of deterioration of fish (26). Although it is generally assumed that few risks are associated with yeasts in terms of the production of BAs (27–29), some strains of different yeast species have already been described as BA producers (30–33). Thus, the objective of this work was to evaluate *in vitro* the safety characteristics of 20 strains belonging to *Saccharomyces* and non-*Saccharomyces* with good probiotic potential, thereby discarding those with adverse effects on health in food industry applications.

## Materials and Methods

### Yeast Strains

Twenty yeast strains, both *Saccharomyces* and non-*Saccharomyces* (*Pichia, Kluyveromyces, Hanseniaspora, Candida*, and *Zygosaccharomyces*), were studied (Table 1). They were isolated from different food ecosystems in Castilla-La Mancha. The non-*Saccharomyces* had been identified up to strain level in previous studies (2). All of them were conserved at −80°C in the culture collection of the Yeast Biotechnology Laboratory (University of Castilla-La Mancha, Spain) and, before using, a fresh culture on YPD agar (Yeast Peptone Dextrose) (Scharlau) at 30°C/24 h was obtained.

**Table 1:** Code number and origin of the studied yeasts.

**Specie**	**Strains**	**Origin**
*Saccharomyces boulardii*	24	Ultra Levura®^*^
*Saccharomyces cerevisiae*	3, 6, 7, 95, 127, 128, 132, 137, 139, 146	Winery
*Pichia kudriavzevii*	1003, 1200	Winery
*Lachancea thermotolerans*	1039	Winery
*Hanseniaspora osmophila*	1056, 1094	Winery, distillery
*Candida vini*	1063	Winery
*Pichia anomala*	1082, 1090	Distillery
*Zygosaccharomyces bailii*	1213	Pickles

* *Saccharomyces boulardii probiotic preparation*.

The strains were selected for this study according to their good probiotic potential *in vitro*, shown in previous works (2, 4). *Saccharomyces boulardii* CNCM I-745 was used as a positive control, since it is the only commercial probiotic yeast. All the assays were performed in triplicate.

A bacterial culture of *Staphylococcus aureus* (CECT 86) was used as positive control in some virulence factors.

### Typing of *Saccharomyces* Strains

The genetic variability of the *Saccharomyces* isolates and the commercial probiotic yeast was evaluated by PCR amplification (in a Perkin–Elmer GeneAmp PCR System 2400) of inter-δ region with primer pair δ2 (5′-GTGGATTTTTATTCCAAC-3′)–δ12 (5′-TCAACAATGGAATCCCAAC-3′) and δ12–δ21 (5′-CATCTTAACACCGTATATGA-3′) following the protocol described by Legras and Karst (34).

δ-PCR products were analyzed by electrophoresis in 1.5% agarose gel, according to the standard procedure for identifying at strain level.

### Antibiotic Resistance

Antibiotic resistance was carried out using Liofilchem© antibiotic strips. Erythromycin (0.016–256 mg mL^−1^), Gentamicin (0.064–1,024 mg mL^−1^), Chloramphenicol (0.016–256 mg mL^−1^), Tetracycline (0.016–256 mg mL^−1^), and Ampicillin (0.016–256 mg mL^−1^) were used. The strips containing the antibiotics were placed onto the surfaces of YPD Agar plates inoculated with the different yeasts (10^6^ cfu mL^−1^). The system was incubated at 30°C for 24 h. This was a modified E-test, thus the presence of a halo indicated inhibition of the target test. The shape of the halo was an ellipse and the intersection of the lower part of the ellipse with the test strip indicated the MIC value (Minimal Inhibitory Concentration).

### Antifungal Resistance

Resistance to certain common antifungals was evaluated. The methodology used was as follows. The yeasts were seeded on Petri dishes using the microbial turf technique and then a drop of 5 μL of each antifungal solution was added. The plates were incubated at 30°C for 48 h. After this time, the halo around the drops indicated yeast inhibition.

The sensitivity of the strains against the antifungal compounds was determined by measuring the diameter (mm) of total (complete absence of growth) and partial (slight growth) inhibition zones.

The antifungals used were Nystatin (21 mg mL^−1^) (*Mycostatin*®, Bristol-Myers Squibb, S.A.), Ciclopirox-Olamine (1 mg mL^−1^) (*Cyclochem*®, Ferrer), Clotrimazole (10 mg mL^−1^) (*Canesten*®, Bayer), and Fluconazole (150 mg mL^−1^) (*Fluconazole*®, Cinfa).

### Biogenic Amine Production

Before analysis, with the aim of promoting enzyme induction, 6 passes were made from the preculture of each strain in YPD broth supplemented with 0.1% (w/v) of each precursor amino acid (L-histidine monohydrochloride, L-ornithine monohydrochloride, tyrosine disodium salt and L-lysine monohydrochloride) (Sigma, St. Louis, USA) and 0.005% (w/v) of pyridoxal-5-phosphate at 30°C for 24 h. Then the cultures (10^6^ cfu mL^−1^) were centrifuged (15,000 × g, 5 min, 4°C) and cell-free supernatants were used for the assay. A non-inoculated medium was used as a control.

Quantification of 7 BAs (histamine, tyramine, putrescine, cadaverine, tryptamine, 2-phenylethylamine, and spermidine) was carried out by RP-HPLC using a diethyl ethoxymethylenemalonate (DEEMM) derivatization method (35). The samples stored at −20°C were thawed and immediately derivatized. For that purpose, a mixture of 1 mL of the sample, 1.75 mL of 1 mol/L borate buffer pH 9.0, 750 μL of methanol and 30 μL of DEEMM was incubated at 30°C in an ultrasonic bath for 30 min. The samples were then heated at 70°C for 2 h to allow the complete degradation of excess DEEMM and reagent by-products. After derivatization, the samples were filtered through regenerated cellulose esters 0.2 μm membranes (Análisis Vínicos, Tomelloso, Spain) coupled to a syringe into conical vials.

The analysis was performed using an Agilent 1200 HPLC (Agilent Technologies, Madrid, Spain) equipped with a Zorbax Eclipse XDB C18 column particle size 5 mm (250 mm × 4.6 mm), an Agilent guard cartridge C18 particle size 5 mm (12.5 mm × 4.6 mm) and a photodiode array detector (Agilent Technologies, Madrid, Spain). The mobile phase consisted of a 50 mmol L^−1^ acetate buffer pH 8.75 with 0.02% sodium azide (eluent A) and an 80:20 mixture of acetonitrile and methanol (eluent B). Samples (50 μL) were applied to the column and eluted at a flow rate of 0.9 mL min^−1^ according to the binary gradient shown in Table 2. The target compounds were identified by their retention times and their spectral characteristics at 280 nm and were quantified using the external standard method. The limits of quantification for the 7 amines were: histamine: 0.13 mg L^−1^; tyramine: 0.07 mg L^−1^; putrescine: 0.03 mg L^−1^; cadaverine: 0.03 mg L^−1^; tryptamine: 0.13 mg L^−1^, 2-phenylethylamine: 0.07 mg L^−1^, and spermidine: 0.20 mg L^−1^.

**Table 2:** Binary elution gradient for HPLC determination of aminoenone derivatives of biogenic amines.

Time (min)	0.0	12.0	16.0	21.0	23.0	25.0	28.0	30.0
Eluent A (%)	72	72	28	18	0	0	72	72
Eluent B (%)	28	28	72	82	100	100	28	28

### Bile Salts Deconjugation Activity

The BSH activity was determined as described by Du Toit et al. (36), with slight modifications. Fresh yeast cultures (10^6^ cells mL^−1^) were streaked in triplicate on YPD agar containing 0.5% (w/v) taurodeoxycholic acid sodium salt (TDCA; Sigma, USA) and 0.037% (w/v) CaCl_2_. The plates were incubated at 37°C for 72 h.

For qualitative assaying of the deconjugation activity, the formation of an opaque or whitish halo zone around the biomass due to the release of free bile acids was observed (37).

### Enzymatic Activities

#### Coagulase

The coagulase activities were assessed using a classical tube test according to Yigit et al. (38). From the overnight culture of each strain, a suspension was prepared in YPD broth with 10^6^ cells mL^−1^, and then 0.1 ml was inoculated into a tube containing 0.5 ml of BBL™ Coagulase Plasma, Rabbit, with Citrate (Fisher Scientific). The tubes were incubated at 30°C and observed for clot formation at 2, 4, 6, and 24 h.

The degree of clot formation was rated from (+) to (++++), according to the different reactions, where “+” is small, unorganized (threadlike) clots; “++” consists of a small, organized clot; “+++” is represented by a large, well-formed clot (moves when the tube is inverted); and “++++” is denoted by a firm clot which remains in place when the tube is inverted (38). On the contrary, the non-formation of any type of clot indicates that the microorganism does not possess said activity. *S. aureus* (CECT 86) was reported as a positive control for coagulase expression.

#### Deoxyribonuclease (DNase)

To detect extracellular DNase production, plates of DNase Test Agar were used with and without methyl green (VWR International Eurolab S.L. and MAIN S.L., Barcelona, Spain), according to Sánchez and Colom (15). From the overnight culture of each strain, a suspension was prepared in sterile PBS with a population of 10^6^ cells mL^−1^ and then 5 μL were added in drops to the plates which were incubated at 30°C for 7 days.

The presence of DNase was indicated by the formation of clear halos around the colonies. In the plates without methyl green, halos were revealed with the addition of HCl (1N). As a positive control, a strain of *S. aureus* (CECT 86) was used.

#### Hemolysis

The hemolytic activity of the yeasts was determined using the procedure described by Yadav et al. (39) with some modifications. A suspension with 10^6^ cells mL^−1^ in sterile PBS was prepared from the overnight culture of each strain and then 5 μL were added onto Trypticase Soy Agar plates, supplemented with 5% (w/v) sheep's blood (VWR International Eurolab S.L., Barcelona, Spain). They were incubated at 30°C (40) and checked after 48 h to examine β-hemolysis, α-hemolysis or non-hemolytic activities. If the surrounding medium contained any shades of brown or green, the “hemolysis” was considered “alpha,” whereas if, in the area around the colony there was a clear zone tending toward the color and transparency of the base medium, the hemolysis was considered “beta.” On the other hand, if a reaction was not observed in the surrounding medium, it indicated the lack of hemolysis (41). A strain of *S. aureus* (CECT 86) was used as a positive control.

#### Protease and Phospholipase

A protease production assay was performed as described by Llopis et al. (21). The test medium consisted of agar plates containing bovine serum albumin (BSA) (pH 5), supplemented with 0.02% (w/v) MgSO_4_ × 7H_2_O (Panreac, Barcelona, Spain), 0.25% (w/v) K_2_HPO_4_ (Panreac, Barcelona, Spain), 0.50% (w/v) NaCl (Panreac, Barcelona, Spain), 0.10% (w/v) dried yeast extract (Pronadisa, Madrid, Spain), 2% (w/v) glucose (Panreac, Barcelona, Spain), 0.25% (w/v) BSA (Fraction V, Sigma, USA), and 2% (w/v) agar.

Regarding the phospholipase production assay, the protocol outlined by Llopis et al. (21) was followed. Lipolytic activity was determined with an egg yolk medium. The test medium was prepared with Sabouraud Dextrose Agar (Difco), 5.85% (w/v) NaCl (Panreac, Barcelona, Spain), 0.06% (w/v) CaCl_2_ (Panreac, Barcelona, Spain), and 10% (v/v) sterile egg yolk (Pronadisa, Madrid, Spain).

In both assays, a suspension from the overnight culture of each strain was prepared in sterile PBS with a population of 10^6^ cells mL^−1^ and then 5 μL were seeded on the respective agars. The plates were incubated at 30°C for 4 days to search for proteinase, and for 7 days to search for phospholipase.

**Figure 1:**
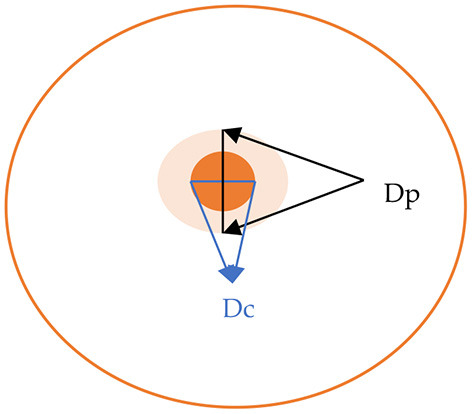
Calculation of the Pz value for enzymatic activity, Pz = Dc/Dp (Dc, diameter of colony; Dh, diameter of colony plus precipitation zone).

The activity was visualized as an area of precipitation around the biomass of each strain. The value of enzymatic activity (Pz) was expressed as the ratio of the colony diameter alone, to the colony diameter plus the precipitation zone, as described in Figure 1. Therefore, the level of activity was indicated as: negative activity (Pz = 1), low activity (Pz = 0.99–0.70), moderate activity (Pz = 0.69–0.50), and high activity (Pz ≤ 0.50).

### Statistical Analysis

A one-way analysis of variance (ANOVA) was applied to the BA results as well as comparing the Pz values of yeast isolates in the secretion of the hydrolytic enzymes studied, using the Student-Newman-Keuls (S-N-K) test for comparison of the means (*P* < 0.05). All the statistical analyses were performed using the IBM SPSS statistics package ver. 24.0 (SPSS Inc., Chicago, IL, USA).

## Results and Discussion

### Typing of *Saccharomyces* Strains

**Figure 2:**
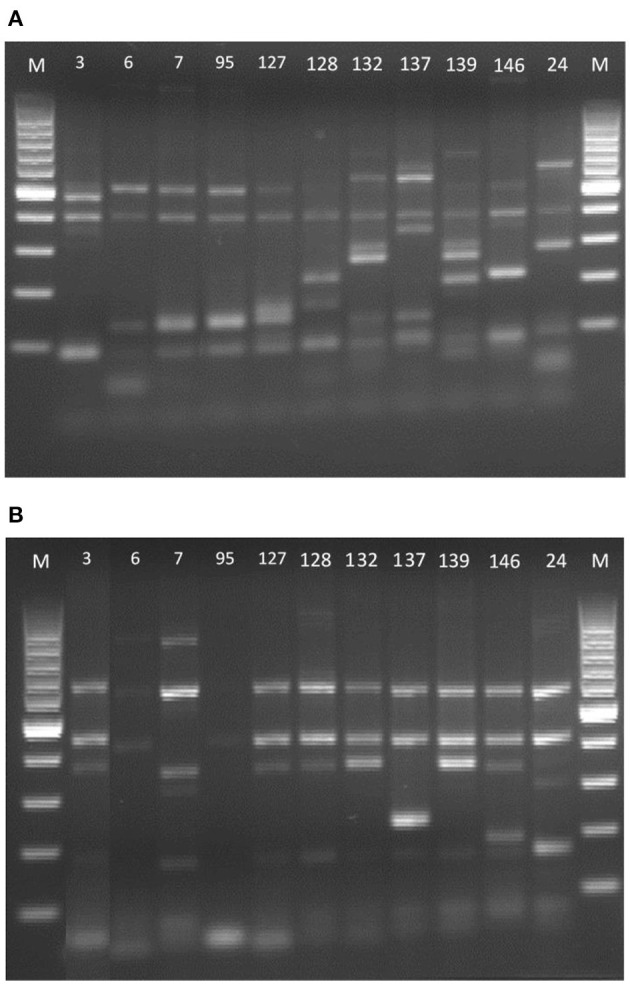
δ-PCR amplification patterns of the DNA of the Saccharomyces strains. **(A)** Primer pair δ2–δ12 and **(B)** primer pair δ12–δ21. 100-bp DNA ladder marker (Biotools, Madrid, España) served as the size standard for both.

As can be seen in Figure 2A, when amplifying the inter-δ region with the primer pair δ2-12, strains 7, 95 and 127 were apparently the same, but when using the primers δ12–δ21, the genetic profiles were different. The same occurred with strains 3, 127, 128, 132, and 139 whose bands obtained with the primers δ12–δ21 (Figure 2B) were very similar, but with the primer pair δ2–δ12 it was observed they were not the same strains. Therefore, combining both results, it was demonstrated that the 10 *S. cerevisiae* of the present study (isolated from different wineries) are distinct from each other and that they also have different profiles from the strain marketed as the only probiotic yeast (26).

### Antibiotic and Antifungal Resistance

All yeasts evaluated showed resistance to the antibiotics studied. The same data are shown by other authors (42). On the contrary, none of the 20 yeasts showed resistance to any of the antifungal products used in this study (Nystatin, Ciclopirox-Olamine, Clotrimazole, and Fluconazole) except *H. osmophila* (1056) which, in contact with Ciclopirox-Olamine, did not show any halo of inhibition.

**Figure 3:**
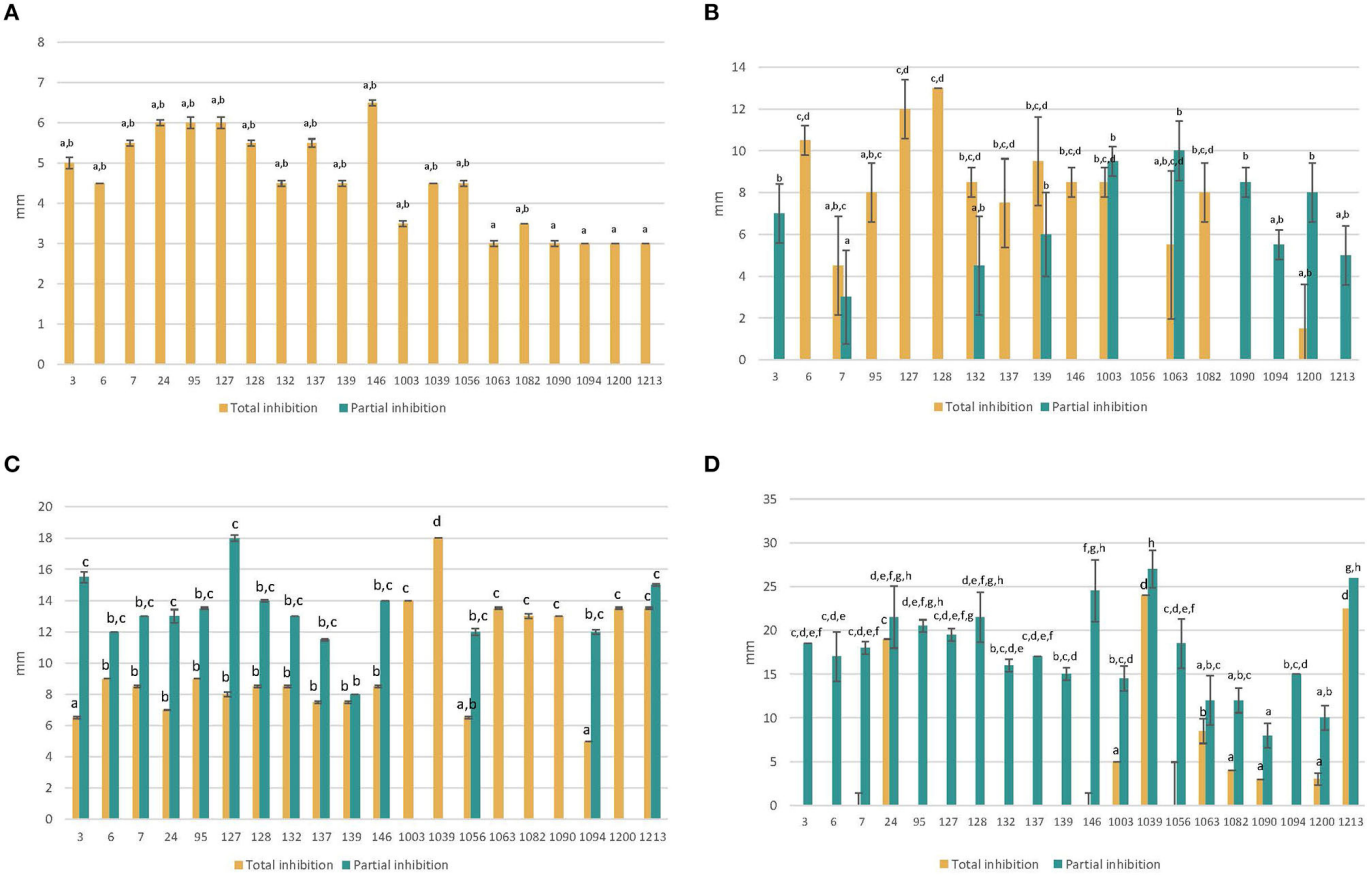
Total and partial inhibition of each strain against Nystatin **(A)** and Ciclopirox-Olamine **(B)** and Clotrimazole **(C)** and Fluconazole **(D)** (mean values ± standard deviation; *n* = 3). ^a−*h*^Different letters indicate significant statistical differences (*p* < 0.05) between strains (for total and partial inhibition) according to the S-N-K test from ANOVA.

**Figure 4:**
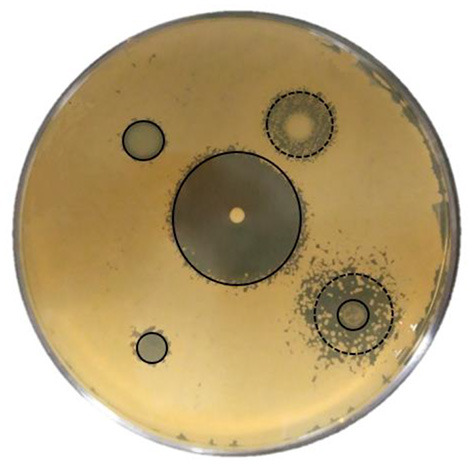
Halos of growth inhibition of a yeast against antifungals assayed. **—**, total inhibition; **—**, partial inhibition.

The results of the inhibition halo tests with the antifungal compounds are shown in Figure 3. In the case of Nystatin and Ciclopirox-Olamine, the strains with the highest resistance, and therefore the smallest halo of inhibition, were non-*Saccharomyces*, unlike what occurred with Clotrimazole and Fluconazole, for which the *Saccharomyces* strains were the most resistant. Nystatin and Clotrimazole were able to totally inhibit the growth of all the strains, although the latter was the most effective. In contrast, Fluconazole showed the lowest effect and only inhibited a total of 8 strains, with the rest showing a partial effect. In all cases, the strains showed significant (*P* < 0.05) differences between them. On the other hand, taking into account the global antifungal effect, it was observed that the most resistant strains were *S. cerevisiae* 3 and *H. osmophila* 1094, while the most sensitive strain was *L. thermotolerans* 1039 (Figure 4 shows a clear example of total and partial inhibition).

These results agree with those reported by Abulreesh et al. (43). Maciel et al. (44) also observed how different non-*Saccharomyces* yeasts were susceptible to antifungals, including those of the *Pichia* genus which was susceptible to Fluconazole.

### Biogenic Amine

Seventeen of the 20 yeast strains evaluated in this study can be considered as non-amine or very low-amine producers (<1 mg L^−1^, Table 3). Among the four remaining strains, *P. kudriavzevii* 1200, *P. anomala* 1090, and *Z. bailii* 1213 produced mainly histamine in concentrations below 23 mg L^−1^ and with significant (P < 0.05) differences between them. On the other hand, the *H. osmophila* 1094 strain accounted for the highest total BA value, mainly due to a very high production of tyramine (almost 700 mg L^−1^) and also to a considerable amount of 2-phenylethylamine (nearly 65 mg L^−1^). It was the only strain which produced these two amines and it also produced histamine in a lower quantity (8.45 mg L^−1^). It is considered that, in terms of good fermentation practices and safe consumption, a fermented food should not exceed 200 mg kg^−1^ of total BAs (45). Among the strains evaluated, all the yeasts except *H. osmophila* 1094 meet this criterion (they produce <200 mg Kg^−1^ of total amines).

**Table 3:** Concentrations (mg L^−1^) of biogenic amines produced by yeasts strains (mean values ± standard deviation; *n* = 3).

	**Strain**	**Histamine**	**Tyramine**	**Putrescine**	**Tryptamine**	**Cadaverine**	**2-phenylethyamine**	**Spermidine**	**Total BAs**
*C. vini*	1063	–	–	0.20^a,b^ ± 0.03	–	0.49^a^ ± 0.08	–	–	0.69^a^ ± 0.10
*H. osmophila*	1056	–	–	–	–	0.33^a^ ± 0.13	–	–	0.33^a^ ± 0.13
	1094	8.45^a^ ± 0.27	696.69 ± 3.34	–	–	0.42^a^ ± 0.10	64.72 ± 1.56	–	765.62^e^ ± 0.83
*L. thermotolerans*	1039	–	–	0.27^a,b^ ± 0.06	–	0.57^a^ ± 0.12	–	–	0.83^a^ ± 0.18
*P. anomala*	1082	–	–	–	–	0.48^a^ ± 0.09	–	–	0.48^a^ ± 0.09
	1090	12.71^c^ ± 1.10	–	0.23^a,b^ ± 0.10	–	0.50^a^ ± 0.11	–	–	13.44^c^ ± 1.19
*P. kudriavzevii*	1003	–	–	–	–	–	–	–	–
	1200	22.37^d^ ± 2.22	–	0.24^a,b^ ± 0.05	–	0.48^a^ ± 0.05	–	–	23.09^d^ ± 2.22
*S. boulardii*	24	–	–	0.16^a^ ± 0.07	–	0.41^a^ ± 0.07	–	–	0.57^a^ ± 0.14
	3	–	–	–	–	0.32^a^ ± 0.15	–	–	0.32^a^ ± 0.15
	6	–	–	–	–	–	–	–	–
	7	–	–	–	–	0.30^a^ ± 0.08	–	–	0.30^a^ ± 0.08
	95	–	–	–	–	–	–	–	–
*S. cerevisiae*	127	–	–	0.39^c^ ± 0.13	–	–	–	–	0.39^a^ ± 0.13
	128	–	–	0.31^b,c^ ± 0.11	–	0.45^a^ ± 0.16	–	–	0.75^a^ ± 0.26
	132	–	–	0.23^a,b^ ± 0.04	–	0.40^a^ ± 0.09	–	–	0.63^a^ ± 0.12
	137	–	–	–	–	–	–	–	–
	139	–	–	–	–	0.30^a^ ± 0.15	–	–	0.29^a^ ± 0.15
	146	–	–	–	–	–	–	–	–
*Z. bailii*	1213	11.53^b^ ± 0.85	–	0.21^a,b^ ± 0.14	–	0.49^a^ ± 0.16	–	–	12.20^b^ ± 0.59

a−e *Different letters in the same column indicate significant statistical differences (P < 0.05) according to the Student-Newman-Keuls test from ANOVA*.

These results show that BA production was proven as a strain-specific property, as has been previously reported for bacterial strains (46, 47). The production of histamine has been reported for *Candida* and *Pichia* strains in concentrations below 5 mg L^−1^ (48). Kung et al. (31) found 6 histamine-producing yeast strains belonging to the *Candida glabrata* and *Candida rugosa* species, capable of producing from 9.2 to 41.7 mg L^−1^ of histamine. Chang et al. (32) reported a strain of *Zygoascus hellenicus* var. *hellenicus* capable of producing amounts of histamine (14.6 mg L^−1^) comparable to those obtained in this study. On the other hand, the production of tyramine by strains of *Kluyveromyces lactis* (205 mg L^−1^) and *Yarrowia lipolytica* (198 mg L^−1^) has been reported (48), these values being much lower than those produced by strain 1094 of *Hanseniaspora osmophila* in the present work. The production of 2-phenylethylamine by yeasts has been reported for the different yeast strains of *Kloeckera, Metschnikowia, Brettanomyces, Candida, Rhodotorula*, and *Trichosporon* (49, 50). However, in all cases, the concentrations were much lower than those produced by the *H. osmophila* 1094 strain in the present work.

### Bile Salts Deconjugation Activity

The results of the virulence factors studied are shown in Table 4. It was observed that 55% of the yeasts showed BSH activity. The 4 strains of the *Pichia* genus (1003, 1200, 1082, and 1090) were positive, unlike those corresponding to *Hanseniaspora* (1056 and 1094). Regarding *S. cerevisiae*, not all the strains presented the same trend, with some of them being positive and others being negative. The results obtained were also unrelated to their origin. The commercial probiotic turned out not to show any activity.

**Table 4:** Summary table of virulence factors studied in the different yeasts.

	**Strain**	**BSH**	**DNAse with methyl green**	**DNAse without methyl green**	**Hemolysine**	**Coagulase**	**Protease**	**Phospholipase**
*C. vini*	1063	–	NG	–	–	–	+++	–
*H. osmophila*	1056	–	NG	–	–	–	+++	–
	1094	–	NG	–	–	–	+++	+++
*L. thermotolerans*	1039	+	NG	–	–	–	+++	–
*P. anomala*	1082	+	NG	–	–	–	+++	+++
	1090	+	NG	–	–	–	+++	+++
*P. kudriavzevii*	1003	+	NG	–	–	–	+++	–
	1200	+	NG	–	–	–	+++	–
*S. boulardii*	24	–	–	–	–	–	+++	–
	3	+	–	–	–	–	+++	+++
	6	+	–	–	–	–	+++	+++
	7	–	–	–	–	–	+++	+++
	95	–	–	–	–	–	+++	+++
*S. cerevisiae*	127	+	–	–	–	–	++	+++
	128	+	–	–	–	–	+++	+++
	132	–	–	–	–	–	+++	–
	137	–	–	–	–	–	+++	–
	139	+	–	–	–	–	+++	+++
	146	–	–	–	–	–	+++	+++
*Z. bailii*	1213	+	NG	–	–	–	+++	–

The results were not related between genders, nor were correlations found with other studies. Tanaka et al. (51) already reported that the molecular weight and structure of BSH is strain dependent. Istiqomah et al. (52) found BSH activity in 104 yeasts (from among 112 isolated and tested) belonging to *Saccharomyces, Candida*, and *Cryptococcus*, while others like Sourabh et al. (53) found no activity in any of the 23 yeast isolates tested which had been obtained from various traditional fermented foods of the Western Himalayas.

Deconjugation has been included by the World Health Organization (WHO) experts as one of the main activities of intestinal microorganisms (14) since probiotics could maximize their survival prospects in the hostile environment of the gastrointestinal tract. In addition, this activity has been correlated with the reduction of the cholesterol level in the blood (23). However, it is not yet completely clear whether BSH activity is a desirable property, since large amounts of deconjugated bile salts may have undesirable effects for the human host (54).

### Coagulase Activity

The coagulation test was performed on citrated rabbit plasma and showed that all yeasts gave negative results, being unable to coagulate the rabbit plasma at the incubation times tested (2, 4, 6, and 24 h) at 37°C (Table 4), unlike *S. aureus* CECT 86 (positive control) which was able to coagulate it in the first hour.

This shows the difference from yeast pathogenic strains, such as *Candida albicans*, which do show positive activity (55, 56) like other *Candida* species isolated from patients with a pathology (56). No data was found in the literature on coagulase activity in any other strains except those of the *Candida* genus.

### Deoxyribonuclease Activity

Deoxyribonuclease activity (DNAse) was studied in two different culture media. In DNAse agar medium with methyl green, all strains belonging to the *Saccharomyces* genus were able to grow but none showed enzymatic activity. Non-*Saccharomcyes* yeasts did not grow in this media. In DNase agar without methyl green, all the yeasts grew although none of them showed hydrolysis halos with the addition of hydrochloric acid (Table 4). Only the *S. aureus* strain, used as a positive control, could grow and showed a hydrolysis halo in both media, although it was more intense in the medium without methyl green.

Although there is little information about DNAse activity in yeasts, the strains with this activity have been isolated from clinical environments and immunosuppressed patients, for example *Cryptococcus neoformans* and *Cryptococcus gattii* (15) or *C. albicans* (16, 38). Investigation of DNAse activity in yeast isolates requires standardization and technique perfection in order to improve sensitivity in the detection of the activity and mechanisms of action of this enzyme (40).

### Hemolysin Activity

None of the 20 strains studied showed this activity in TSA-blood medium after 48 h although all of them were able to grow.

Hemolysin expression is believed to be an inherent factor that is triggered under specific conditions (57). In yeasts, hemolytic factor is known to be a putative virulence factor contributing to pathogenicity in the *Candida* species (57). Furthermore, only certain strains of this genus, isolated from patients with pathologies, have been reported to produce this activity (58, 59).

### Proteolytic and Phospholipase Activity

**Figure 5:**
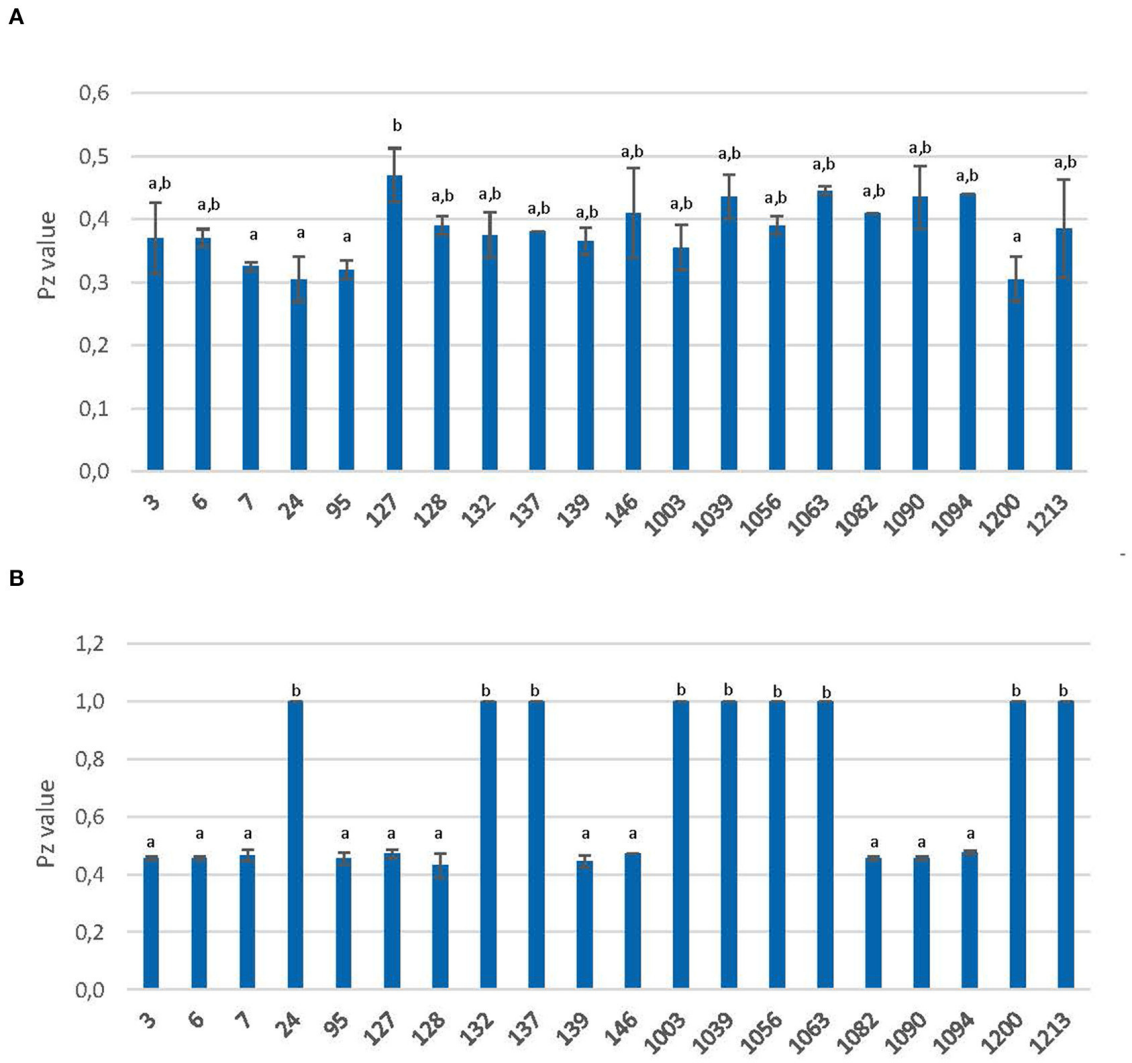
Extracellular secretion of protease **(A)** and phospholipase **(B)** yeast strains. Activity is expressed as Pz value (mean values ± standard deviation; *n* = 3). ^a,b^Different letters indicate significant statistical differences (*p* < 0.05) between strains according to the S-N-K test from ANOVA.

Extracellular secretion of proteases and phospholipases were analyzed by measuring the precipitation area around each colony after growing on BSA and Egg-Yolk media, respectively. The qualitative results and Pz values are shown in Table 4 and Figure 5.

All yeasts were able to grow on BSA agar and showed high proteolytic activity (Pz ≤ 0.50) with significant (*P* < 0.05) statistical differences (Figure 5A). Strain 127 showed the lowest value (0.47), with significant (*P* < 0.05) differences from the rest. On the other hand, strains 7, 95, and 1200 showed the highest (*P* < 0.05) values along with the commercial probiotic yeast (strain 24). The average Pz value for this activity was 0.38.

Moradi et al. (60) also found proteolytic activity in *Saccharomyces* sp. and *Kluyveromyces* sp. (isolated from sweet fruit and dairy samples). Ombrella et al. (61) detected that more than 60% of the *C. albicans* studied showed a high degree of proteolytic activity. De Llanos et al. (20) and Llopis et al. (21) showed that most *Saccharomyces* isolates from dietary supplements also present this activity. In the case of Jafari et al. (17), who evaluated pathogenic strains, high proteolytic activity was observed in addition to coagulase activity, unlike the strains studied here that did not present this last activity.

The results for phospholipase activity showed that all the studied strains could grow in this medium but 9 of them showed no activity (Pz = 0.50). Therefore, according to the S-N-K test from ANOVA, two groups were clearly differentiated: those with a Pz-value of 0.43–0.48 and on the other hand, those with a Pz value of 1 (Figure 5B).

De Llanos et al. (20) carried out a comparative study between clinical and industrial *S. cerevisiae* yeast strains based on the various phenotypic traits associated with pathogenicity. They showed that, like the pathogenic strains, those isolated from food sources as well as *S. boulardii* secreted high levels of protease and phospholipase, which coincides with the results obtained in the present study. Therefore, neither activity could be considered a relevant aspect in determining whether yeasts are safe or not. Similarly, Llopis et al. (21) found yeasts included in dietary supplements with phospholipase activity levels ranging from low to moderate. *S. boulardii* was included in the low activity group, unlike in the present work where it showed no activity.

## Conclusions

Probiotics should be non-pathogenic microorganisms, safe for consumption. Wild yeasts from food environments represent a valid source of potential probiotics but security evaluation should be systematically included in searching for them.

According to the results of the present study, it can be said that most of the yeasts of this work are safe microorganisms. Several groups could be considered, depending on the values of different assays: strains with BSH and phospholipase activity (*S. cerevisiae* 3, 6, 127, 128, and 139 and *P. anomala* 1082), strains with BSH but without phospholipase activity (*L. thermotolerans* 1039, *P. kudriavzevii* 1003), strains without BSH but with phospholipase activity (*S. cerevisiae* 7, 95, 146) and strains without either of them (*C. vini* 1063, *H. osmophila* 1056, *S. cerevisiae* 132 and 137), including the commercial probiotic *S. boulardii*. All the strains included in the different groups can be inhibited by any of the antifungals tested.

The only strain discarded for use in the food industry was *H. osmophila* 1094, due to its high production of BAs, especially tyramine (almost 700 mg L^−1^). All the rest could be proposed for probiotic applications as a valid alternative to the widely available probiotic *yeast S. boulardii*, as well as in fermentation processes in the food industry. Further investigation is needed to clearly define their health-promoting efficacy, as well as the appropriate dosage, following the criteria of the WHO and EFSA recommendations.

## Data Availability Statement

The original contributions presented in the study are included in the article/supplementary material, further inquiries can be directed to the corresponding author/s.

## Author Contributions

JP and MA-V: conceptualization, validation, investigation, and visualization. MF-G, JP, and MA-V: methodology, writing—review and editing, and funding acquisition. PF-P, IR, and MF-G: formal analysis and resources. PF-P and IR: data curation. PF-P: writing—original draft preparation. MA-V: supervision and project administration. All authors have read and agreed to the published version of the manuscript.

## Conflict of Interest

The authors declare that the research was conducted in the absence of any commercial or financial relationships that could be construed as a potential conflict of interest.
